# Supplementary figures and images for: GWAS in a Collection of Bulgarian Old and Modern Bread Wheat Accessions Uncovers Novel Genomic Loci for Grain Protein Content and Thousand Kernel Weight
Source: Plants (Basel). 2024 Apr 12;13(8):1084. doi: 10.3390/plants13081084 (PMC11054703; doi:10.3390/plants13081084)

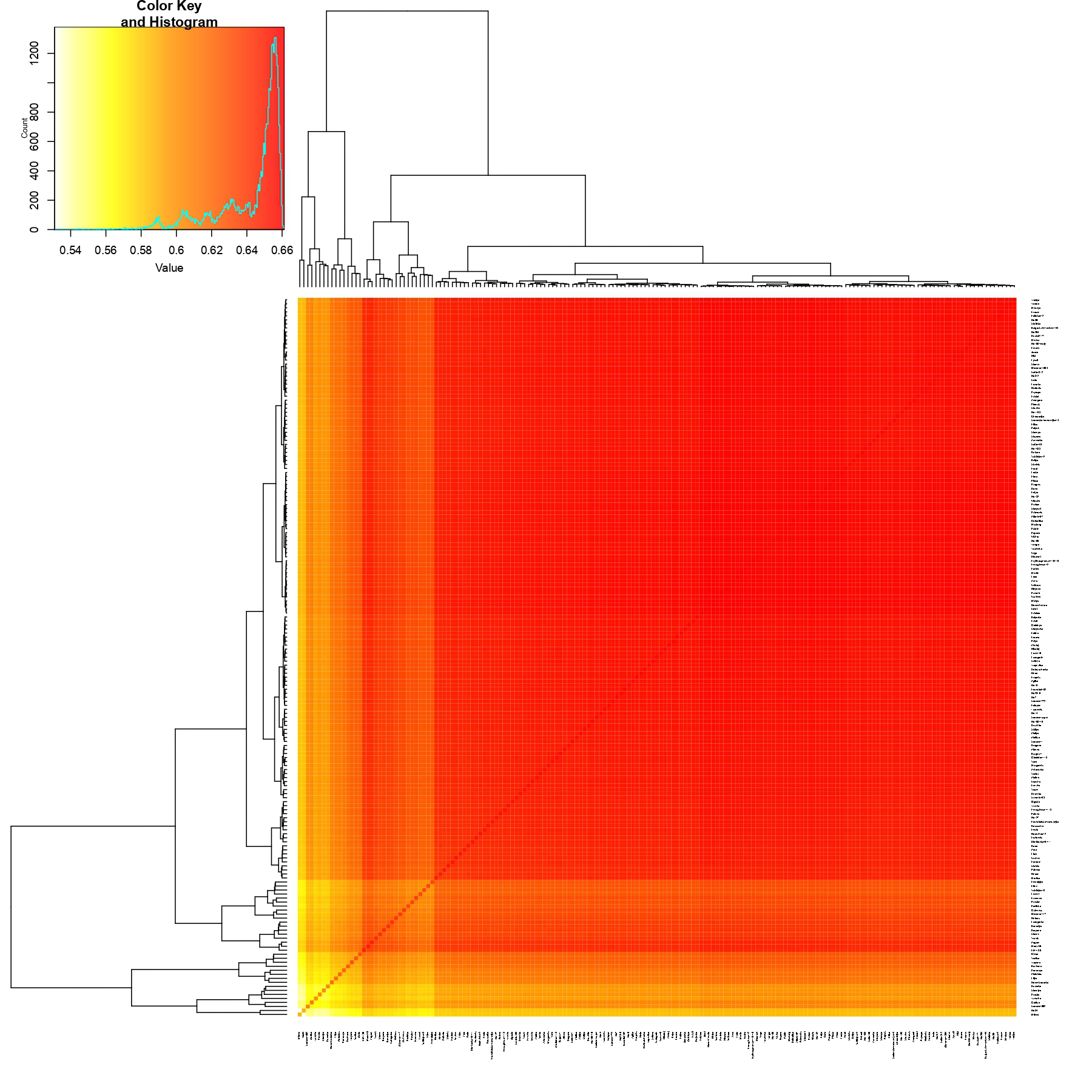

Supplement: Supplementary file 1 [file plants-13-01084-s001.zip › Figure-S1.jpg]

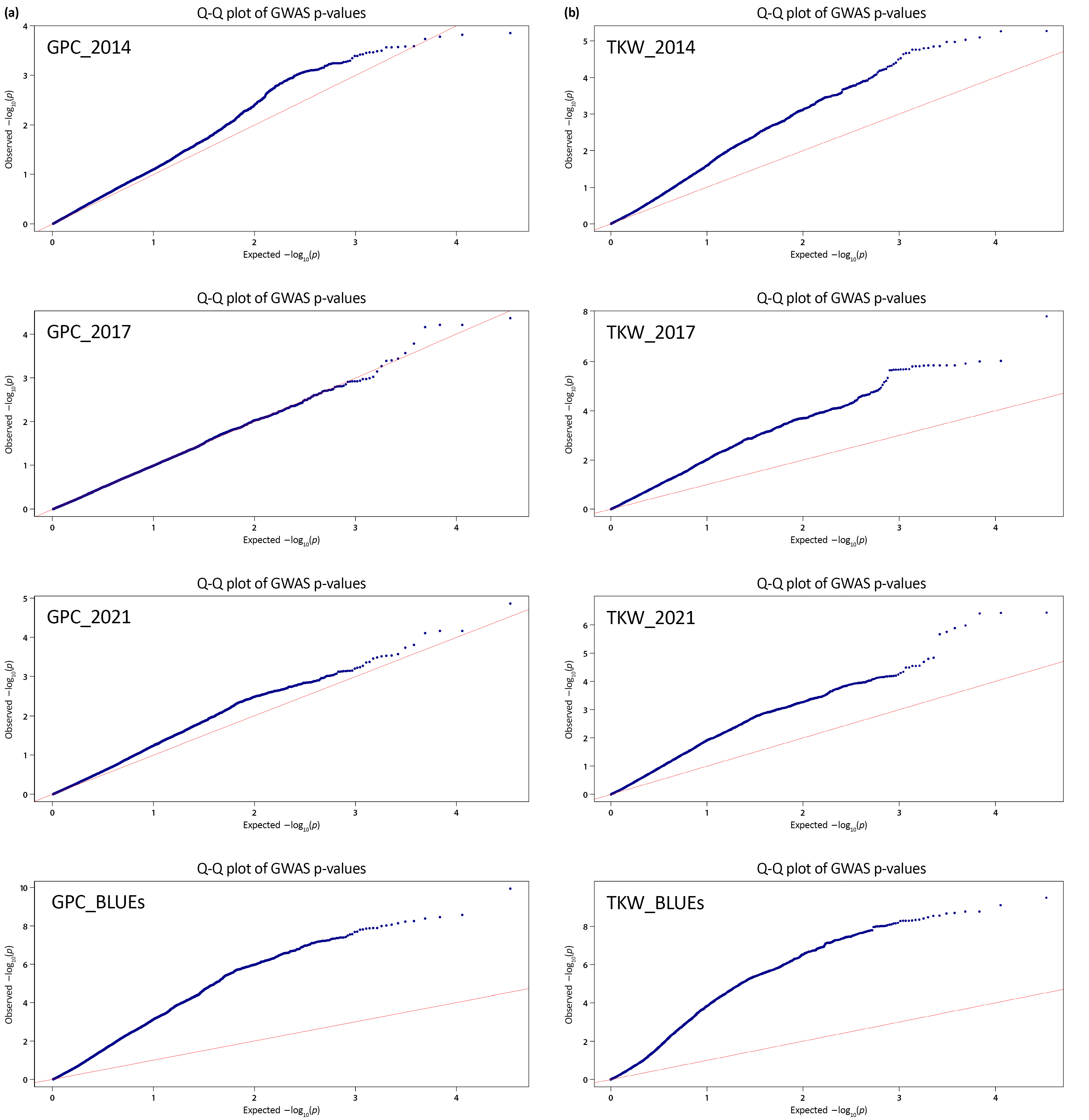

Supplement: Supplementary file 1 [file plants-13-01084-s001.zip › Figure-S2.jpg]

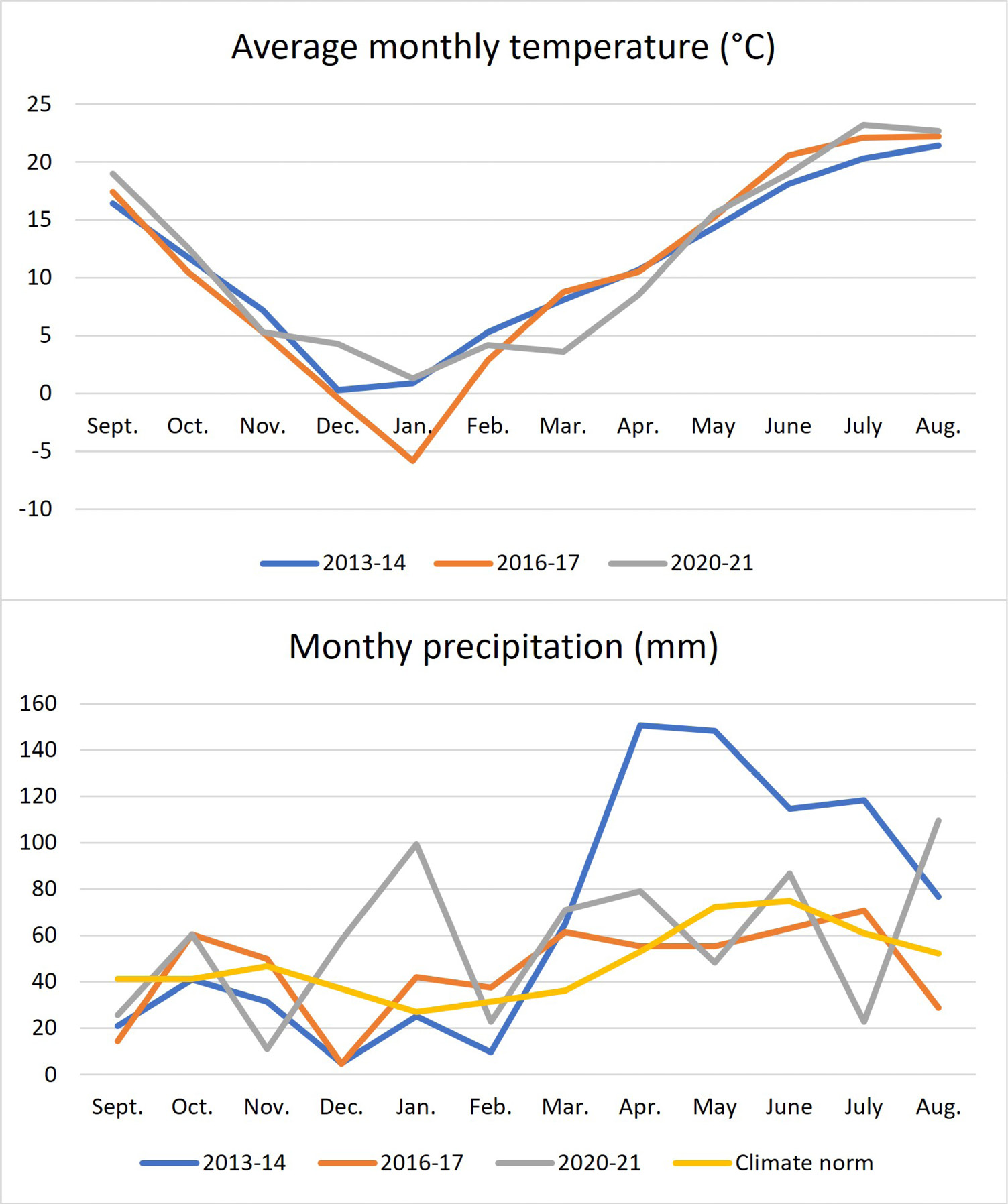

Supplement: Supplementary file 1 [file plants-13-01084-s001.zip › Figure-S3.jpg]
